# Patient dosimetry for ${}^{90}Y$ selective internal radiation treatment based on ${}^{90}Y$ PET imaging

**DOI:** 10.1120/jacmp.v14i5.4371

**Published:** 2013-09-06

**Authors:** Sherry C. Ng, Victor H. Lee, Martin W. Law, Rico K. Liu, Vivian W. Ma, Wai Kuen Tso, To Wai Leung

**Affiliations:** ^1^ Department of Clinical Oncology Queen Mary Hospital Hong Kong; ^2^ Department of Clinical Oncology University of Hong Kong Hong Kong; ^3^ Department of Diagnostic Radiology Queen Mary Hospital Hong Kong

**Keywords:** yttrium‐90 microsphere, liver cancer, PET, dosimetry, LQ model

## Abstract

Until recently, the radiation dose to patients undergoing the ${}^{90}Y$ selective internal radiation treatment (SIRT) procedure is determined by applying the partition model to ${}^{99m}\text{Tc}$ MAA pretreatment scan. There can be great uncertainty in radiation dose calculated from this approach and we presented a method to compute the 3D dose distributions resulting from ${}^{90}Y$ SIRT based on ${}^{90}Y$ positron emission tomography (PET) imaging. Five ${}^{90}Y$ SIRT treatments were retrospectively analyzed. After ${}^{90}Y$ SIRT, patients had ${}^{90}Y$ PET/CT imaging within 6 hours of the procedure. To obtain the 3D dose distribution of the patients, their respective ${}^{90}Y$ PET images were convolved with a Monte Carlo generated voxel dose kernel. The sensitivity of the PET/CT scanner for ${}^{90}Y$ was determined through phantom studies. The 3D dose distributions were then presented in DICOM RT dose format. By applying the linear quadratic model to the dose data, we derived the biologically effective dose and dose equivalent to 2 Gy/fraction delivery, taking into account the spatial and temporal dose rate variations specific for SIRT. Based on this data, we intend to infer tumor control probability and risk of radiation induced liver injury from SIRT by comparison with established dose limits. For the five cases, the mean dose to target ranged from 51.7 ± 28.6Gy to 163 ± 53.7 Gy. Due to the inhomogeneous nature of the dose distribution, the GTVs were not covered adequately, leading to very low values of tumor control probability. The mean dose to the normal liver ranged from 21.4 ± 30.7 to 36.7 ± 25.9 Gy. According to QUANTEC recommendation, a patient with primary liver cancer and a patient with metastatic liver cancer has more than 5% risk of radiotherapy‐induced liver disease (RILD).

PACS number: 87.53.Bn

## I. INTRODUCTION

Selective Internal Radiation Treatment (SIRT) is the delivery of radiation treatment via intrahepatic arterial administration of ${}^{90}Y$ microspheres. ${}^{90}Y$ SIRT is an emerging modality in the management of patients with inoperable primary and metastatic liver cancer. It is based on the unique pattern of hepatic blood flow by which the majority of the tumor blood supply is derived from the hepatic artery, whereas hepatic parenchymal blood flow largely comes from the portal vein. When ${}^{90}Y$ microspheres are introduced through the hepatic artery, ${}^{90}Y$ microspheres will preferentially localize in the peritumoral and intratumoral arterial vasculature, delivering high dose of radiation to the tumors. The use of ${}^{90}Y$ microspheres for treatment of liver neoplasms has been around for some time; prospective clinical trials have shown encouraging response and survival data.^(1)^

Until now, the radiation dose for the SIRT procedure was estimated by the partition method based on pretreatment ${}^{99m}\text{Tc}$ MAA diagnostic angiogram.^(^ ^2^ ^,^ ^3^ ^)^ However, there are great uncertainties associated with this approach. The reproducibility of catheter positioning during therapy is questionable and the particle size of ${}^{90}Y$ microsphere is different from that of ${}^{99m}\text{Tc}$ MAA, so the distribution of ${}^{99m}\text{Tc}$ MAA may not truly correspond to that of ${}^{90}Y$ microsphere.

In radionuclide therapy, the absorbed dose is usually estimated, because *in vivo* dose measurement is not feasible in clinical settings. However, without accurate patient dosimetry, it is impossible to establish fundamental dose‐response relationship for treatment efficacy and toxicity. Also, it is difficult for different institutes to compare treatment results. For SIRT treatments, there is great inhomogeneity of ${}^{90}Y$ concentration within the patient's body and, because of the short range of the ${}^{90}Y$ beta particles, the total dose and dose rate vary spatially over the irradiated volume. The dose rate also changes temporally as the ${}^{90}Y$ decays. The spatial and temporal variations of dose rate will have different radiobiological effect on the tumor and normal tissues. The partition method assumes a uniform distribution of ${}^{90}Y$ microsphere, disregarding the spatial and temporal variation of the dose and dose rate. Consequently, the radiobiological effects specific to SIRT are ignored, and the partition method is at best a simplified picture for the patient dosimetry.

${}^{90}Y$ has an internal pair production component with a branching ratio of $31.87\;\pm \;0.47\;\times \;10^{-6}$.^(4)^ Recently, this pair production component of ${}^{90}Y$ decay for PET imaging has been employed to evaluate the distribution of ${}^{90}Y$ for patients treated with SIR‐Spheres.^(5)^ The image quality of ${}^{90}Y$ PET is superior to the traditional Bremsstrahlung SPECT and correlates well to the diagnostic ${}^{18}\text{FDG}$ PET and CT scan. In this paper, we computed patient dose from ${}^{90}Y$ SIR‐Spheres based on high‐quality ${}^{90}Y$ PET images. This makes possible the calculation of accurate 3D physical dose distributions.

Considering the radiobiological effect of varying dose rates, tumor control probability and normal liver dose constraints should not be directly applied to the physical dose data. We used the linear quadratic (LQ) model to convert the physical dose to the biologically effective dose (BED). The BED was then converted to dose equivalent to delivery at 2 Gy/fraction (EQ2), which is the typical dose per fraction used in conventional external beam radiation therapy and upon which published limits for tumor control and normal tissue complication are usually based. With EQ2, the application of dose limits to tumor control and normal tissue are meaningful.

## II. MATERIALS AND METHODS

### A. Absorbed dose from ${}^{99m}\text{Tc}$ MAA scintigraphy

From January 2011 to March 2012, five patients (all male, aged 45–78 years) who received SIRT had subsequent ${}^{90}Y$ PET scan in our institute. Three patients had inoperable hepatocellular carcinoma, the other two (patients 1 and 3) had inoperable metastatic liver cancer from primary colorectal cancer. All patients met the manufacturer's assessment criteria for SIRT. Diagnostic whole body ${}^{18}\text{FDG}$ PET/CT was done to assess the tumor extent and target localization. Prior to SIRT, arteriography, followed by ${}^{99m}\text{Tc}$‐labeled MAA scintigraphy, was performed on all patients to assess the hepatic arterial vasculature and also for hepatopulmonary shunt fraction calculation. It also detected any gastrointestinal reflux, if present. Whole body anterior and posterior planar images of the chest and abdomen were taken after infusion of 4 mCi of ${}^{99m}\text{Tc}$ MAA. Based on the ${}^{99m}\text{Tc}$ MAA distribution, absorbed dose was calculated with the partition method. The tumor absorbed dose, $D_{t}$, and normal liver absorbed dose, $D_{l}$, can be calculated from Eq. (1) and (2):^(2)^

(1) $$D_{t}=49.8\times \frac{A(\text{GBq})}{M(\text{kg})}\times \frac{r}{1+(M_{t}/M)(r-1)}$$

(2) $$D_{1}=49.8\times \frac{A(\text{GBq})}{M(\text{kg})}\times \frac{1}{1+(M_{t}/M)(r-1)}$$

Here, *A* is the administered activity, *M* is the mass of the liver, $M_{t}$ is the mass of tumor, and *r* is the tumor/liver activity uptake ratio determined from the scintigraphy. The tumor/liver activity uptake ratio, r, was calculated as (counts from tumor ROI)/(counts from normal liver ROI).

### B. Calibration of PET/CT Scanner for ${}^{90}Y$

**Figure 1:**
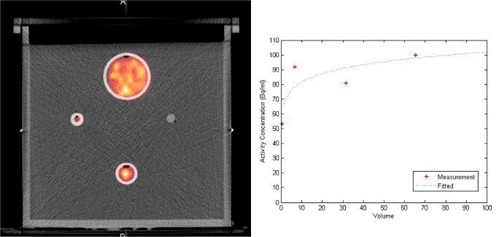
PET/CT image (a) of the calibration phantom. Variation (b) of the observed mean activity concentration for each spherical insert with the volume of the inserts.

To obtain the activity concentration of ${}^{90}Y$ within the patients' bodies, sensitivity of the PET scanner for ${}^{90}Y$ was determined. Calibration of the PET/CT scanner for the ${}^{90}Y$ isotope was performed with a rectangular phantom containing four spherical inserts containing ${}^{90}Y$ solution in a cold background. ${}^{90}Y$ solution was used instead of ${}^{90}Y$ SIR‐Spheres because the SIRT spheres tend to settle over time during imaging, resulting in an uneven distribution of ${}^{90}Y$ within the spherical inserts. This could affect calibration accuracy. The inserts were filled with a nominal activity concentration of 2.996 MBq/ml of ${}^{90}Y$. The volume of the spheres ranged from $0.22\;{\text{cm}}^{3}$ to $65.45\;{\text{cm}}^{3}$, whereas the background volume was 8.26 liters. (See Fig. 1 for the PET/CT image of the calibration phantom.)

### C. Dosimetry from ${}^{90}Y$ PET/CT imaging

${}^{90}Y$ PET/CT imaging was performed with GE Discovery STE PET/CT scanner (GE Healthcare, Waukesha, WI) within 6 hours of the SIRT procedure. Attenuation corrected ${}^{90}Y$ PET images were collected at two bed positions 30 minutes each, covering the whole liver and lung base. For a known activity distribution in a homogeneous tissue of infinite extend, the spatial dose distribution can be determined by convolving the nuclide concentration (from the PET images in our case) with the dose point kernel, described in the following:^(6)^

(3) $$\dot{D}(r)=A(r)⊗k(r)$$

Here D(r) is the absorbed dose rate at point r, A(r) is the cumulative activity at point r, and k(r) is the dose point kernel. The Medical Internal Radiation Dose (MIRD) Committee calculated S values at the voxel level for several relevant radionuclides using EGS4.^(7)^ In this study, we used the published S values presented in MIRD Pamphlet 17^(7)^ of 6 mm voxels for ${}^{90}Y$ in a homogeneous medium.

**Figure 2:**
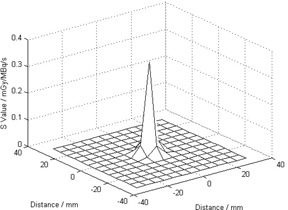
Resampled voxel dose kernel to match the voxel size of the PET images.

**Figure 3:**
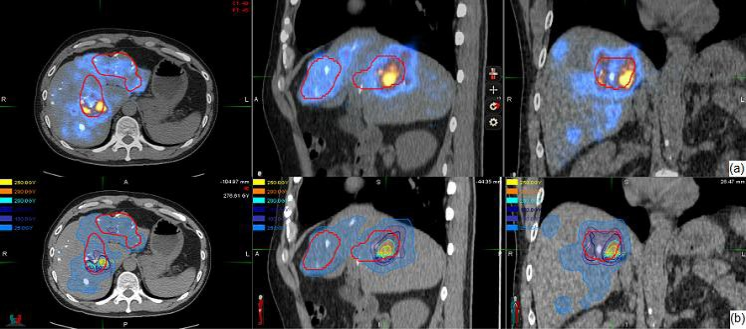
Resampled voxel dose kernel to match the voxel size of the PET images.

Based on the calibration, the observed count rate for each voxel on the PET images was converted to the activity concentration for the ${}^{90}Y$ distribution. The voxel size of the PET images was $5.47\;\times \;5.47\;\times \;3.27\;{\text{mm}}^{3}$, therefore the 6 mm voxel point dose kernel from Bolch et al.^(7)^ was first resampled to voxel size equivalent to the PET image voxel size for convolution. Figure 2 shows the two‐dimensional representation of the resampled voxel point dose kernel which was subsequently convolved with the PET images to compute the instantaneous dose rate. The three‐dimensional convolution was done in MATLAB (The MathWorks, Natick, MA; www.MathWorks.com). The resultant values were again resampled to voxel size of $2.5\;\times \;2.5\;\times \;2.5\;{\text{mm}}^{3}$ to match the voxel size of the CT images. Then by integrating the decaying dose rate to infinity, we obtained the total dose delivered to each voxel. DICOM‐RT dose files were generated corresponding to the dose values. (See Fig. 3 for the dose distribution (Fig. 3(a)) and ${}^{90}Y$ PET/CT image (Fig. 3(b)) of patient 1).

For DVH computation, the target and organ at risk need to be localized. For each patient, the liver was localized on the ${}^{90}Y$ PET/CT images. The target was localized on the diagnostic ${}^{18}\text{FDG}$ PET/CT images by radiation oncologists. The diagnostic ${}^{18}\text{FDG}$ PET/CT images were registered to the ${}^{90}Y$ PET/CT images and target copied to the ${}^{90}Y$ PET/CT images for DVH computation.

### D. Computation of biologically effective dose and dose equivalent to 2 Gy per fraction delivery

Once injected into the hepatic artery, the microspheres are distributed preferentially in the tumor areas, being trapped within the tumor microvasculature. There is very little biological clearance and ${}^{90}Y$ decays with its physical half‐life of 2.67 days. The ${}^{90}Y$ microspheres become permanent implants. There are two considerations in applying the physical dose calculated from above to assess tumor control and the risk of radiotherapy‐induced liver disease (RILD). Firstly, the delivery dose rate is changing, starting at maximum at the embolization procedure (can reach 4 Gy per hour for our group of patients), exponentially dropping to almost zero in ten half‐lives. Secondly, the total dose and the dose rate are inhomogeneous throughout the target volume and organ‐at‐risk (OAR) volume. Thus the biological effect of the dose is also varying throughout the target volume and the OAR. It will be incorrect to compare the physical dose computed from the ${}^{90}Y$ PET images directly with published data for tumor control and risk of RILD, when published data for radiation dose of tumor control and radiation‐associated injury is often derived from external beam therapy data, most likely in fractionation of 2Gy/fraction.

The biologically effective dose (BED) is defined in terms of both physical and radiobiological parameters, and is a measure of the total amount of lethal damage sustained by a specific tissue. The BED is obtained by multiplying the total physical dose with a modifying factor which takes into account the physical aspects of dose delivery (dose rate, dose per fraction). For a permanent implant delivering an initial dose rate of $R_{o}$, and involving a nuclide with decay constant X, Eq. (4) gives the BED.^(8)^ Here μ is the time constant for sublethal damage repair.^(9)^ Since the ${}^{90}Y$ microspheres are trapped in the capillary bed, there is virtually no biological clearance and ${}^{90}Y$ decays with its physical half‐life of 2.67 days, $X\;=\;0.2596\;d^{-1}$. $\mu \;=\;\text{log}2/T_{1/2}$ is the repair constant for the repair of sublethal damage. Repair $T_{1/2}$ for tumors and normal tissues are less well established than α/β, and from Gerbaulet et al.,^(10)^ $T_{1/2}\;=\;30$ min to 1 h for early‐reacting normal tissues and tumors, and $T_{1/2}\;=\;1.5\;h$ for late‐reacting normal tissues. We used $T_{1/2}\;=\;1\;h$ for tumor and $T_{1/2}\;=\;1.5\;h$ for healthy liver in our study.

(4) $$\text{BED}=\frac{R_{O}}{\lambda }\left(1+\frac{R_{O}}{\left(\mu +\lambda )(\alpha +\beta \right)}\right)$$

The initial dose rate $R_{o}$ is given by total dose divided by the decay constant X.

Making use of this equation and the differential dose‐volume histogram (dDVH), we converted the physical dose dDVH to the BED dDVH. Then the BED dDVH was converted to equivalent doses as if given as fractionated irradiation at 2Gy/fraction by Eq. (5). Here we were using α/β = 2.5 Gy for normal liver and α/β = 10 Gy for tumor, values taken from Gerbaulet et al.:^(10)^

(5) $$\text{nd}{=}^{\text{BED}}{/}_{\left(1+\frac{d}{\alpha /\beta }\right)}$$

where *nd* is the total dose corresponding to dose delivered at d = 2 Gy fractions. We denoted this dose as EQ2.

### E. Computation of Tumor Control Probability

To calculate the overall tumor control probability based on the differential volume histogram, for each dose bin of the dDVH, of volume $V_{i}$ receiving a dose $D_{i}$, with clonogenic density $p_{i}$; the tumor control probability can be written as:^(11)^

(6) $${\text{TCP}}_{i}=\text{exp}[-\rho_{i}V_{i}\text{exp}(-{\alpha D}_{i})]$$

Here, a is the sensitivity of the tumor cells to radiation.

Then the total tumor control probability can be found by multiplying the individual TCPs together, and so:

(7) $$\text{TCP}=\prod_{i}[\text{exp}-\rho_{i}V_{i}\text{exp}(-{\alpha D}_{i})]$$

In Eq. (7), a equals to $0.33\;{\text{Gy}}^{-1}$,^(12)^ and we assumed a value is constant (all the tumor cells have the same sensitivity). The clonogenic density $\rho_{i}$ is equal to $10^{7}$.^(13)^

## III. RESULTS

Sensitivity of our PET scanner for the ${}^{90}Y$ isotope was determined by phantom study. Figure 1(a) shows the calibration phantom and Fig. 1(b) is the variation of the observed mean activity concentration for each spherical insert with the volume of the inserts. The quality of the PET/CT image of the phantom was excellent, even the $0.22\;{\text{cm}}^{3}$ sphere was visible. Each sphere was contoured and average activity concentration determined. The average activity concentrations were plotted against the volume of spheres and result showed in Fig. 1(b). The dotted line showed the fitted data. Sensitivity of our PET scanner was found to be 0.32 cps/MBq.

From December 2010 to March 2012, five patients had SIRT treatment in our institution with ${}^{90}Y$ PET imaging done within 6 hours after SIRT. Patients 2 and 3 suffered from metastatic liver cancer, patients 1, 4, and 5 had hepatocellular carcinoma. Figure 3(a) shows the ${}^{90}Y$ PET image of patient 1 who suffered from inoperable multifocal hepatocarcinoma. 1.3 GBq of ${}^{90}Y$ SIR‐Spheres (SIRTeX Medical Limited, Sydney, Australia) was administered via a Fr3 Progreat microcatheter (Terumo Medical Corp., Somerset, NJ) inserted into Fr5 Yashiro catheter (Terumo Medical Corp.) at the hepatic proper artery. Then ${}^{90}Y$ PET imaging was performed 5 hours afterwards. PET images showed the presence of hot spots in areas close to or within the target delineated by the radiation oncologist on the diagnostic ${}^{18}\text{FDG}$ PET/CT images. Based on the ${}^{90}Y$ PET images, the dose to the patients was computed. Figure 3(b) shows the resultant isodose distribution. For this patient, the mean dose to the target and normal liver was 112.5 ± 47.6 Gy and 25.8 ± 28.1 Gy, respectively.

**Figure 4:**
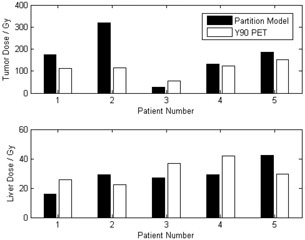
Comparison of dose computed from partition method and ${}^{90}Y$ PET dosimetry for target and normal liver for the five patients in this study. Administered activity of ${}^{90}Y$ is shown in the boxes.

**Figure 5:**
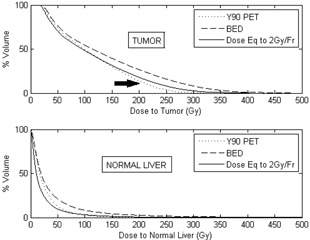
Cumulative DVH for various dose quantities of target and normal liver. The fraction of target volume receiving a higher dose is greater for EQ2 than the physical dose (see arrow), while for the healthy liver, the physical DVH and EQ2 DVH are very similar.

For the five patients, we also computed the dose to target and dose to normal liver based on the partition method using Eqs. (1) and (2). Figure 4 shows a comparison of the dose calculated from the partition method and ${}^{90}Y$ PET‐based patient dosimetry for these five patients. Based on the differential dose‐volume histograms for target and normal liver and using Eqs. (4) and (5), the BED DVH and the EQ2 DVH were computed. Figure 5 shows the DVHs of target and normal liver for patient 1. Table 1 shows the dose to target and normal liver for all the patients.

Using Eq. (7), we tried to compute the TCP for the five patients. A MATLAB program was written to do the computation based on the dDVHs. However, due to the inhomogeneous nature of SIRT, substantial cold spots were found in the target of all the patients and the calculated TCP were effectively zero for all the patients.

**Table 1:** Absorbed dose data for the five patients

		*Mean Dose to Target*				*Mean Dose to Normal Liver*			
*Patient*	*Inj. Act. (GBq)*	$\text{Target Vol}\;({\text{cm}}^{3})$	${}^{90}Y$ *PET (Gy)*	*BED10 (Gy)*	*EQ2 (Gy)*	$\text{Vol}\;({\text{cm}}^{3})$	${}^{90}Y$ *PET (Gy)*	*BED2.5 (Gy)*	*EQ2 (Gy)*
1	1.3	60.7	112.5	139.3	116.1	1540.7	25.8	38.8	21.5
2	1.1	75.7	114.4	140.3	116.9	1070.3	22.3	38.4	21.4
3	1	350.7	56.1	62.0	51.7	1949.2	36.6	54.4	30.2
4	1.2	85.3	122.7	151.7	126.4	1341.7	41.9	66.6	36.7
5	1.2	51.6	152.9	195.6	163.0	1201.2	29.4	52.8	29.4

## IV. DISCUSSION

In this work, we presented a method of patient dosimetry for the SIRT procedure based on ${}^{90}Y$ PET/CT imaging which is practicable in a clinical setting. ${}^{90}Y$ PET is a reliable method for the assessment of SIR‐Sphere distribution with superior image quality compared to ${}^{90}Y$ Bremstrahlung scan. We computed the patients' three‐dimensional dose distributions based on these high‐resolution PET images and represented in DICOM‐RT dose file format. In addition, the CT images provide additional anatomical information so that dose to other structures can be computed as required.

Sensitivity of the PET scanner was determined through phantom study. Sensitivity of our PET scanner for ${}^{90}Y$ was found to be 0.32 cps/MBq. Werner et al.^(14)^ determined the sensitivity of ${}^{90}y$ for their Siemens Biograph 16 HiRez PET scanner at 40 min/bed to be 0.72 cps/MBq, which is comparable to our results. Several studies have used partition model based on ${}^{99m}\text{Tc}$ MAA scans to compute the dose to tumor and normal liver for the ${}^{90}Y$ SIRT procedure.^(^ ^2^ ^,^ ^13^ ^)^ These approaches assumed uniform distribution of activity within each partition and only give an estimate of the dose received in each compartment. One other study has used an approach similar to ours to calculate the radiation absorbed dose for ${}^{90}Y$ SIRT of an HCC patient. Sarfaraz et al.^(15)^ convolved the ${}^{99m}\text{Tc}$ MAA SPECT image with a Monte Carlo generated voxel dose kernel to compute the dose distribution from the ${}^{90}Y$ spheres. However, dosimetry based on ${}^{99m}\text{Tc}$ MAA SPECT imaging has two major drawbacks. First, the difference in particle size of ${}^{99m}\text{Tc}$ MAA from the ${}^{90}Y$ microspheres (MAA particles have diameters in the range 15 to 30 microns and a density of 1.3 g/cc, and SIR‐Sphere microspheres have diameters of 35 ± 5 microns and a density of 1.6 g/cc^(16)^), and thus the distributions of the two types of particles are not identical. Secondly, there is uncertainty arising from catheter placement for ${}^{99m}\text{Tc}$ MAA and ${}^{90}Y$ SIRT which are, in fact, two separate procedures. Our results showed large discrepancies between the doses calculated based on partition method and ${}^{90}Y$ PET dosimetry, especially the dose to the target (difference ranged from ‐53.8% to +178.4%). For the tumor, partition method generally overestimated the dose. Other than the fundamental differences between these two dose calculation methods, the large discrepancy for target dose arises from inconsistency of target localization. The partition method relies on the accuracy of the tumor to liver uptake ratio, r. For our study, this ratio is determined from planar ${}^{99m}\text{Tc}$ MAA scintigraphy for which the tumor and liver were localized on the 2D images. On the other hand, the target volume for the ${}^{90}Y$ PET dosimetry is based on diagnostic ${}^{18}\text{FDG}$ PET/CT. Disagreement of target localization between the two approaches is to be expected, resulting in considerable difference in dose to target for the two methods.

The SIR‐Spheres are classified by regulators as a brachytherapy medical device, and radioembolization with SIR‐Spheres is effectively permanent implant brachytherapy. As discussed above, there is great dose inhomogeneity due to the way the ${}^{90}Y$ microspheres are introduced into the patient's body, and also due to rapid dose falloff with distance from the sources. As ${}^{90}Y$ decays, the dose rate will also change with time. The spatial and temporal variation of dose rate will have varying radiobiological effects on tumor control and normal liver sparing. The effect of these variations cannot be reflected if only the physical dose is reported. Also, because of the dose gradient, each voxel of the target and healthy liver will be receiving a different total dose at a different time, varying dose rate. Applying the LQ model to the mean dose of the target or healthy liver to get the mean BED is oversimplification. Thus, we applied the LQ model to the differential dose‐volume histogram, calculating the BED and EQ2 dose for each dose bin within which the dose and dose rate is relatively constant. This will give us the BED dDVH and EQ2 dDVH. It was based upon these results that we computed the mean BED and mean EQ2 dose to the target and normal liver for each patient. Table 1 shows the mean physical dose, BED, and EQ2 for the target and normal liver. For the five patients, the mean dose to the target ranged from 51.7 ± 28.6 to 163 ± 53.7 Gy. Due to the inhomogeneous nature of the dose distribution, there were significant geographic misses of the target, leading to very low values of tumor control probability. On the other hand, dose to the normal liver ranged from 21.4 ± 30.7 to 36.7 ± 25.9 Gy. According to QUANTEC recommendation,^(17)^ a patient with primary liver cancer and a patient with metastatic liver cancer has more than 5% risk of radiation induced liver disease.

Based on the DICOM‐RT dose file, it is also possible to compute the dose to other critical structures such as lung, stomach, and duodenum, to assess the risk of pneumonia or gastroduodenal ulcer, as long as these structures are contoured and DVH‐computed.

**Figure 6:**
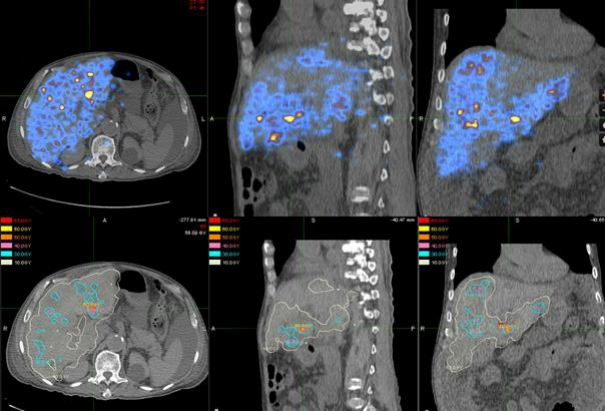
${}^{90}Y$ PET image (top row) 5 hours after treatment for patient 3. There was very little uptake over the whole liver. ${}^{90}Y$ PET based isodose map (bottom row) from PET image.

Strigari et al.^(18)^ reported a mean dose of 110 Gy to target for 73 patients treated at their center with 74% partial or complete response. The mean dose to target for our patients ranged from 51.7 ± 28.6 to 163 ± 53.7 Gy, with median dose equals to 114 Gy. This compares favorably with the Strigari study. Patient #3 has a very low uptake of ${}^{90}Y$ to the tumor and liver. He suffered from extensive liver metastasis from colon cancer and passed away from unrelated complications within one month of the SIRT therapy. Figure 6 shows the ${}^{90}Y$ PET and dose distribution for patient 3. We attempted to compute the TCP for our patients based on Ebert and Hoban.^(11)^ Since there were significant mismatch between the planning target volume and the radioembolization zone, large areas of the PTV received very little dose, resulting in very low TCP values. It is unjustifiable to report the TCP at this stage.

## V. Conclusions

We have presented a method of patient specific dosimetry based on ${}^{90}Y$ PET/CT imaging which is feasible in a clinical setting. Due to inhomogeneity in dose and dose rate from ${}^{90}Y$ throughout the irradiated volume, we propose reporting the BED and the EQ2 dose derived from the physical dose based on the LQ model. Further studies are required to compute the tumor control probability based on the dose distribution. Also a phantom study is needed to validate the dose computation.
